# Separating the Effects of Band Bending and Covalency in Hybrid Perovskite Oxide Electrocatalyst Bilayers for Water Electrolysis

**DOI:** 10.1021/acsami.1c20337

**Published:** 2022-03-16

**Authors:** Lisa Heymann, Moritz L. Weber, Marcus Wohlgemuth, Marcel Risch, Regina Dittmann, Christoph Baeumer, Felix Gunkel

**Affiliations:** † Peter Gruenberg Institute 7, Forschungszentrum Juelich GmbH, 52425 Juelich, Germany; ‡ JARA-FIT, RWTH Aachen University, 52056 Aachen, Germany; § Nachwuchsgruppe Gestaltung des Sauerstoffentwicklungsmechanismus, Helmholtz-Zentrum Berlin für Materialien und Energie GmbH, 14109 Berlin, Germany; ∥ MESA+ Institute for Nanotechnology, Faculty of Science and Technology, University of Twente, 7522 NB Enschede, Netherlands

**Keywords:** oxygen evolution reaction, perovskite oxide, La_1−*x*_Sr_*x*_CoO_3_, Mott−Schottky analysis, covalency, interface hybridization, OER descriptor, band bending

## Abstract

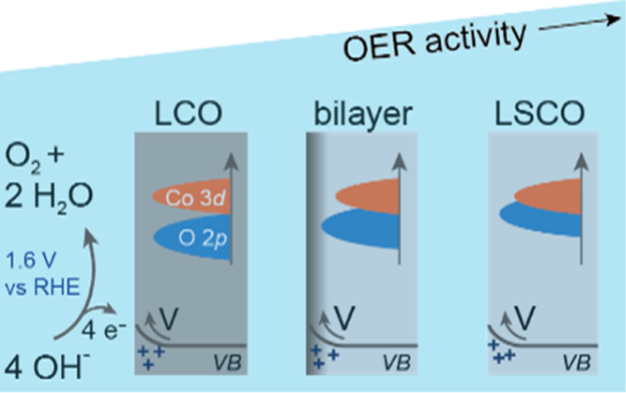

The Co–O covalency in perovskite oxide cobaltites such as La_1–*x*_Sr_*x*_CoO_3_ is believed to impact the electrocatalytic activity during electrochemical water splitting at the anode where the oxygen evolution reaction (OER) takes place. Additionally, space charge layers through band bending at the interface to the electrolyte may affect the electron transfer into the electrode, complicating the analysis and identification of true OER activity descriptors. Here, we separate the influence of covalency and band bending in hybrid epitaxial bilayer structures of highly OER-active La_0.6_Sr_0.4_CoO_3_ and undoped and less-active LaCoO_3_. Ultrathin LaCoO_3_ capping layers of 2–8 unit cells on La_0.6_Sr_0.4_CoO_3_ show intermediate OER activity between La_0.6_Sr_0.4_CoO_3_ and LaCoO_3_ evidently caused by the increased surface Co–O covalency compared to single LaCoO_3_ as detected by X-ray photoelectron spectroscopy. A Mott–Schottkyanalysis revealed low flat band potentials for different LaCoO_3_ capping layer thicknesses, indicating that no limiting extended space charge layer exists under OER conditions as all catalyst bilayer films exhibited hole accumulation at the surface. The combined X-ray photoelectron spectroscopy and Mott–Schottky analysis thus enables us to differentiate between the influence of the covalency and intrinsic space charge layers, which are indistinguishable in a single physical or electrochemical characterization. Our results emphasize the prominent role of transition metal oxygen covalency in perovskite electrocatalysts and introduce a bilayer approach to fine-tune the surface electronic structure.

## Introduction

Electrochemical water splitting receives great scientific, political, and economic attention due to the possibility to store electrical energy from renewable energy sources in the H_2_ chemical bond. The reverse reaction performed in fuel cells gives access to CO_2_-free electricity on demand so that periodic and sudden energy production deficits of wind and solar can be compensated.^1^ Until today, however, the electrochemical water-splitting reaction lacks efficiency at the anode where water is oxidized to O_2_. Currently applied electrocatalysts based on platinum-group metals are expensive and have a low earth abundance, rendering them disadvantageous for a large industrial rollout.^2−4^

Instead, perovskite oxides (ABO_3_) can be designed as effective and low-cost electrocatalysts for electrochemical water splitting by varying the A- and B-site elemental and stoichiometric composition. The B-site is commonly occupied by 3d transition metals such as iron, cobalt, or nickel and can act as catalytically active centers in the alkaline oxygen evolution reaction (OER).^2,5−9^ Perovskite oxides are highly efficient OER electrocatalysts especially when the oxygen site is additionally activated and can participate in the OER mechanism, a phenomenon often denoted as the lattice oxygen-mediated mechanism (LOM).^10,11^

Such an oxygen activation is induced by a high covalency between the transition metal B-site and the oxygen site. The covalency is reflected in the electronic structure by the degree of energetic overlap between oxygen 2p and transition metal 3d states near the Fermi level.^10,11^ The covalency between oxygen and B-site ions can be increased by either choosing a more electronegative transition metal or by changing the oxidation state on the B-site by varying the A-site stoichiometry from a trivalent ion such as La^3+^ to a divalent ion such as Sr^2+^.^10,12^

However, the influence of the covalency on the OER catalytic activity can be entangled with additional changes in electronic properties, such as band bending of the conduction and valence band at the solid/liquid interface after Fermi level equilibration of the electrolyte and perovskite.^13,14^ The resulting space charge layer can affect the electron transport into the electrode.^5,14^ For the anodic reaction on p-type catalyst surfaces, downward band bending under operating conditions would hamper the electron transport from the liquid to the solid phase and cause higher required overpotentials to drive the reaction. In contrast, upward band bending should not limit the reactivity.^5,14^ Especially, low conductivity and low covalency perovskites such as LaCoO_3_ (LCO) are less OER-active catalysts compared to the more covalent La_1–*x*_Sr_*x*_CoO_3,_ where the Sr doping in La_1–*x*_Sr_*x*_CoO_3_ leads to a larger O 2p–Co 3d band overlap and metallic behavior.^10,11,14−16^ It is complicated to differentiate whether the lack in OER activity in LCO is determined by the extent of a space charge layer or by the covalency and therefore by the lower O 2p and Co 3d overlap.^14,17^

In this work, we experimentally distinguish these two phenomena. First, we investigate the O 2p and Co 3d overlap in the near surface by X-ray photoelectron spectroscopy (XPS), and second, we define the band-bending conditions from open-circuit voltage (OCV) toward the OER voltage regime through a Mott–Schottky (MS) analysis. As model catalysts, we compare the highly covalent and OER-active La_0.6_Sr_0.4_CoO_3_ (LSCO) and less covalent and less OER-active LCO since in both perovskites, Co is the only B-site.

To tune the catalytic properties and observe a transition between LCO and LSCO, we designed epitaxial bilayer stacks consisting of LSCO films with LCO capping layers of different thicknesses. Epitaxial thin film growth allows layering the materials (with similar lattice parameters) with unit cell precision on top of each other.^18,19^ This precise thickness control enables positioning the interface of LSCO and LCO in nanometer proximity to the thin film surface so that both layers and their presumed interface hybridization can contribute to the overall OER activity.^20,21^ Previous reports on epitaxially grown SrRuO_3_/SrTiO_3_ stacks revealed hybrid electronic states near the Fermi level that directly impact the OER activity,^20,22^ a scenario that may also occur at the LSCO/LCO interface.

XPS analysis reveals that the near-surface carrier concentration decreases with increasing thickness of the LCO capping layer and that ultrathin LaCoO_3_ capping layers of 2–8 unit cells show increased Co–O covalency compared to single LCO. By conducting the Mott–Schottky (MS) analysis, we could identify that a decreased surface carrier concentration in the LSCO/LCO bilayer structures does not lead to an extended space charge layer that may limit the OER catalytic performance. Instead, the OER activity is dominated by the Co–O covalency and hence by the overlap between the O 2p and Co 3d states. Bilayer stacks of ultrathin LCO capping layers revealed increased Co–O covalency, whereas a thicker LCO capping layer revealed a similar surface valence band electronic structure as single LCO that is also reflected by the OER catalytic activity trend. Our experimental approach thus identifies covalency as opposed to a lack of electronic carriers and disadvantageous band bending as the main driver for intrinsic OER activity trends in La_1–*x*_Sr_*x*_CoO_3_ perovskite oxides.

## Results and Discussion

### Thin Film Growth and Characterization

**Figure 1:**
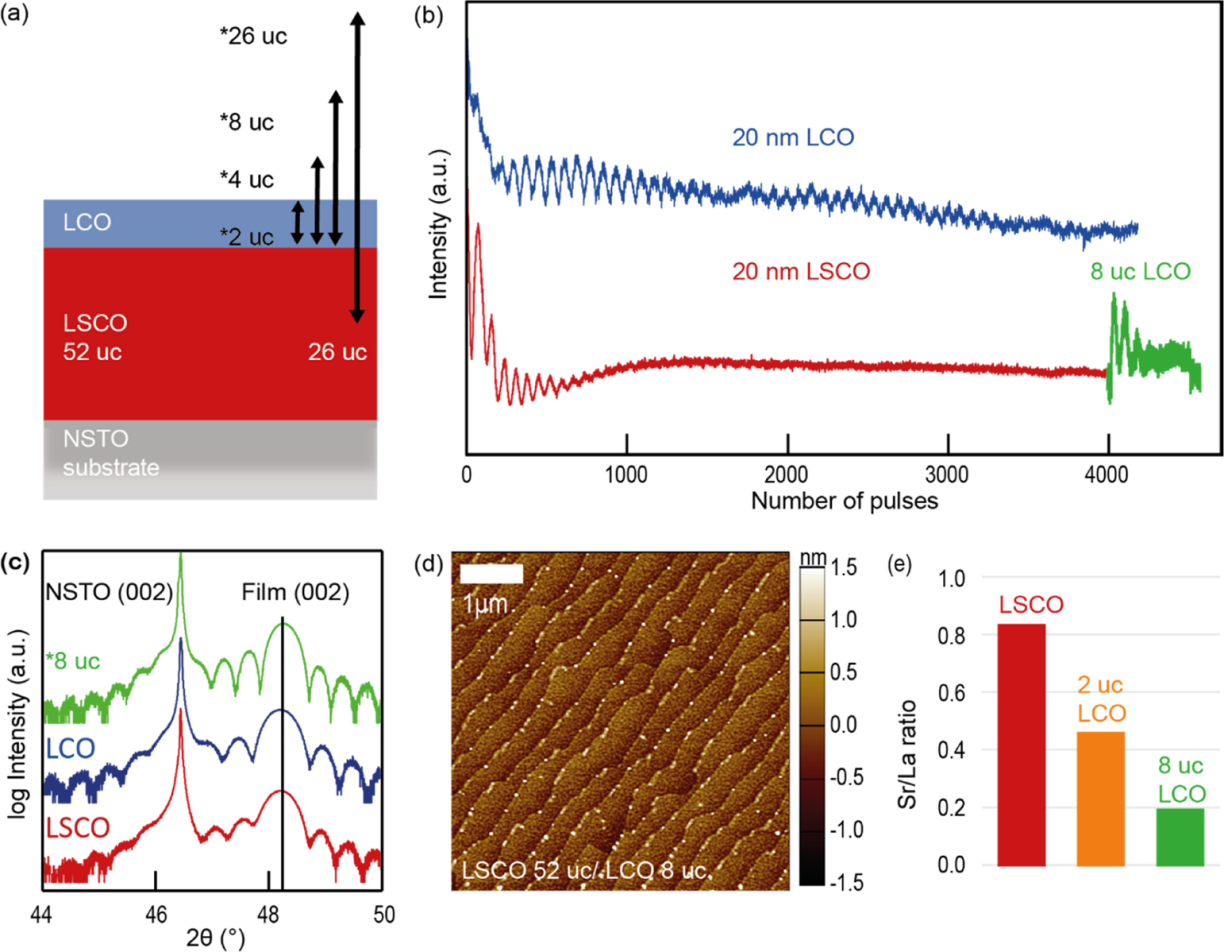
(a) Catalyst design: 52 uc of LSCO were deposited on NSTO by PLD. LCO was deposited subsequently in a thickness of 2, 4, or 8 uc. For a 26 uc LCO layer thickness, the LSCO thickness was decreased to 26 uc. (b) In situ RHEED data of the single LCO 52 uc and LSCO 52 uc and bilayer LSCO 52 uc/LCO 8 uc films. (c) X-ray diffractograms of single LCO 52 uc and LSCO 52 uc and bilayer LSCO 52 uc/LCO 8 uc films. (d) AFM scan of the bilayer sample 52 uc LSCO/8 uc LCO. (e) Sr/La ratio determined from the core-level XPS spectra Sr 3d and La 3d for single LSCO and bilayer samples.

Single LSCO and LCO and bilayer films were fabricated by pulsed laser deposition (PLD) allowing us to grow the films with unit cell precision. First, single LSCO and LCO films were deposited on 0.5 wt % Nb-doped SrTiO_3_ (NSTO) with 20 nm thickness which corresponds to 52 unit cells (uc). Second, the bilayer samples were constructed with a 52 uc-thick LSCO bottom layer and covered with an LCO top layer of 2, 4, and 8 uc thickness (see Figure 1a) to monotonically displace the Sr-containing layer from the surface reducing the total hole carrier concentration in the near-surface region that may influence the band bending during catalysis. Another reference sample comprised a 26 uc-thick LCO layer on LSCO with a reduced layer thickness to 26 uc to keep the overall thickness to about 20 nm.

Tracking the epitaxial growth of LSCO and LCO in situ by reflective high energy electron diffraction (RHEED) (Figure 1b) shows a layer-by-layer growth for LCO over the whole deposition time, whereas LSCO shifts to step-flow-like growth over time.^8^ Subsequent LCO deposition on the LSCO film exhibits two clear oscillations before fading out (PLD growth of the other bilayer films can be seen in Figure S1). A two-dimensional surface is still recognizable after the deposition of the LSCO and LCO films as well as for the bilayer films confirmed by the corresponding RHEED patterns (see Figure S2) and by a smooth, step-terraced surface morphology with minor step-edge decorations (Figures 1d and S1). Clear (002) diffraction peaks of LSCO, LCO, and bilayer films (the bilayer films are represented here by 8 uc LCO on 52 uc LSCO) are detected by X-ray diffraction (XRD) in 2θ–ω measurement geometry (Figure 1c), which indicate a high crystallinity and similar *c*-lattice parameters of the materials. The 52 uc LSCO/8 uc LCO bilayer film is 15% thicker than the 52 uc LSCO and LCO single films and therefore appears with a smaller thin film peak width. Furthermore, pronounced thickness fringes give evidence about the coherent substrate-to-thin film interface and smooth surface of the epitaxial layers.

The XPS analysis in Figure 1e shows a decreasing Sr/La ratio in the near surface with increasing LCO thickness indicating a successful bilayer film growth. The Sr/La ratio of La_0.6_Sr_0.4_CoO_3_ is larger than expected, indicating slight Sr segregation on the surface of the LSCO films.

Co K-edge X-ray absorption spectroscopy (XAS) data revealed a shift of the LSCO absorption spectra toward higher photon energies compared to LCO (Figure S3). This indicates that the average bulk Co oxidation state in LSCO is higher than in LCO due to the divalent Sr ion substitution. The average bulk Co oxidation state in the 52 uc LSCO/8 uc LCO bilayer film is only slightly decreased compared to LSCO as the photon escape depth of >12.9 μm is much larger than the total film thickness of 23 nm (see Figure S3 experimental) so that the top layer contributes little to the probed volume. This is in contrast to the surface-sensitive XPS spectra.

### Electronic Structure Characterization

**Figure 2:**
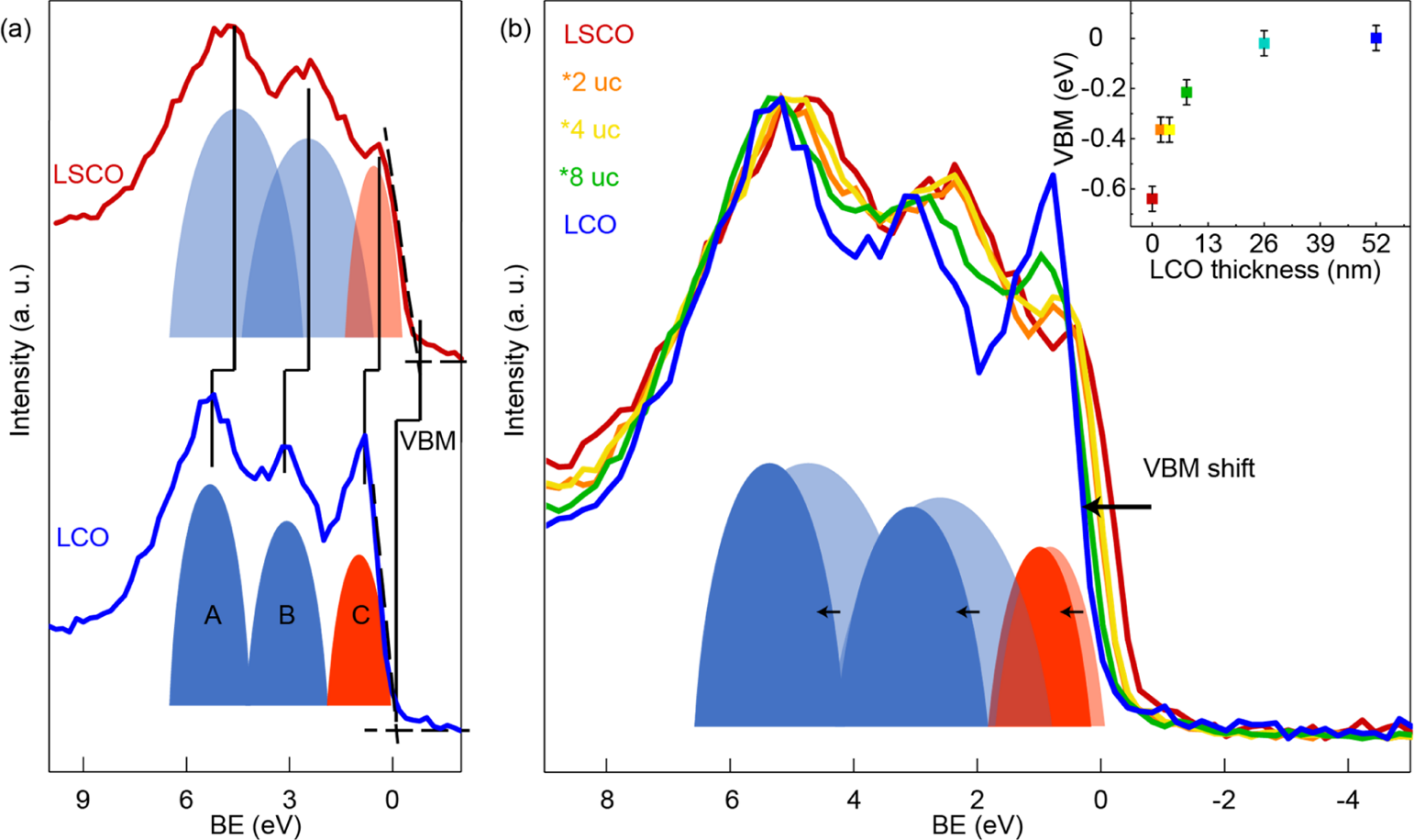
(a) LCO and LSCO XPS of the valence band states close to the Fermi level. LCO and LSCO signals are separated in three main domains as schematically drawn in the spectra with the peak A and B in blue representing two O 2p states and the red peak C representing the Co 3d states.^10,16^ The valence band maximum (VBM) was determined via the zero-photoemission intensity intercept of a linear regression fit of the low-binding-energy edge of the valence band spectra.^23,24^ (b) XPS spectra of the valence states close to the Fermi level of single LSCO and LCO and the bilayer films (2, 4, or 8 uc marked with *) and the single LCO film. Inset: Observed VBM as a function of LCO thickness. The error bars represent the possible maximum deviation of the consecutive measurements.

To evaluate the changes in electronic structure properties of the bilayer catalysts with greater surface sensitivity, we turn to XPS analysis with a mean escape depth of 2.6 nm (ca. 7 uc). We first compare the valence band electronic structure of the two parent compounds LSCO and LCO, as shown in Figure 2a. The electronic states of LCO and LSCO near the Fermi level can be separated into three main contributions, the bonding O 2p states (peak A), the nonbonding O 2p states (peak B), and the antibonding transition metal 3d states (peak C)^16,25^ that are located closest to the Fermi level and therefore determine the valence band maximum (VBM).^26^ As illustrated by Mefford et al. and other research groups, Sr substitution in La_1–*x*_Sr_*x*_CoO_3_ shifts the Fermi level closer to the O 2p states (A and B) and the energy difference between the O 2p and Co 3d states decreases. The higher Co–O energetic overlap described for LSCO compared to LCO is in accordance with our XPS results.^10,11,15^ For the single A-site perovskite LCO, the three main peaks are largely separated in binding energy (Figure 2a bottom). A-site cation substitution in La_0.6_Sr_0.4_CoO_3_ increases the average cobalt oxidation state and moves the Fermi level closer to the O 2p states so that the Co 3d–O 2p binding energy difference decreases (Figure 2a, top).^10,11,15,16^ Furthermore, the strontium substitution goes hand-in-hand with a broadening of the oxygen valence band states since the O 2p orbital environment changes with the presence of additional cations (features A and B become broader as indicated in the sketch in Figure 2a).

These two phenomena, the broadening of the O 2p bands and the smaller binding energy difference, result in a higher overlap of the O 2p and Co 3d band, corresponding to higher covalency of the Co–O bond in LSCO compared to LCO. The three components A, B, and C were fitted in the XPS spectra to quantify their FWHM and peak distance, as shown in Figure S4. Substituting strontium in the LCO lattice also induces degenerative p-type doping for the investigated La_0.6_Sr_0.4_CoO_3_,^16^ as evident in the shift of the VBM to lower binding energies in comparison with the VBM of LCO.

Figure 2b shows the valence band structures of the single LSCO and LCO and bilayer LSCO/LCO thin films. A shift of the VBM is seen in the bilayer sample series from single LSCO to single LCO, meaning that with increased LCO thickness, the surface hole concentration decreases. As can be seen in the inset of Figure 2b, the 26 uc LSCO/26 uc LCO bilayer and single LCO film exhibit almost identical VBM values. As the XPS mean escape depth was 2.6 nm (∼7 uc), the spectrum of the bilayer film is dominated by the 26 uc LCO capping layer. The almost identical VBM value indicates that the surface hole concentration is dominated by the LCO film and the LSCO hole concentration does not contribute to the surface hole concentration anymore.

In addition to the shift of the VBM, the O 2p bands (peaks A and B) shift to higher binding energies and sharpen with the increased LCO thickness which is accompanied by a separation of the Co 3d and O 2p states (Figure 2b). The separation and sharpening of the peaks mainly reflects the ratio change in the detected LSCO and LCO electrons escaping separately from each layer resulting in a superposition of two independent XPS signatures. In addition, hybrid LSCO/LCO energy states contribute to the observed spectral changes because a linear combination model of single LSCO and LCO spectra was insufficient to account for the detailed shape of the real measured bilayer spectra (Figures S5 and S6). The overlap between the O 2p and Co 3d bands is larger for the real spectra compared to the linear combination spectra. This result shows that the LSCO/LCO hybridization leads to an average higher O 2p and Co 3d overlap and hence a higher Co–O covalency in the 2, 4, and 8 uc LCO capping layers compared to the single (or 26 uc-thick) LCO film.

The same trend was confirmed with the XPS spectra of a more surface-sensitive photoemission angle and varied LCO thickness (see Figures S6 and S7). The difference in the original and linear combination spectra is even more apparent in the more surface-sensitive spectra where the superimposed LSCO contribution is even lower and Co 3d–O 2p overlap should be even more determined by a higher LCO character. A more quantitative evaluation of the bilayer film XPS spectra as conducted for single LSCO and LCO in Figure S4 is difficult, since at least six components (reflecting peaks A, B, and C for LSCO and LCO) have to be included in the fitting, resulting in an overdetermination during the fit.

Various processes may contribute to the finally observed hybrid electronic structure and increased Co–O covalency. For one, the LSCO/LCO interfacial hybridization may increase the density of states especially in the region of the Co 3d–O 2p overlap, increasing the Co–O covalency in the LCO capping layer. A similar interfacial hybridization effect was obtained in ultrathin capping SrTiO_3_ layers on SrRuO_3_. The charge redistribution created additional electronic valence states in the band gap of SrTiO_3_ through buried SrRuO_3_.^20^

Besides interface hybridization, the increased covalency in the LCO capping layer can be a result of Fermi level equilibration between LSCO and LCO. The Fermi level in LSCO is closer to the valence band than in LCO,^26^ and therefore, the charge transfer between the two layers may increase the Co oxidation state in the LCO capping layers.^27^ A higher Co oxidation state increases the Co 3d and O 2p overlap and hence the covalency in LCO. Such a correlation of the Co oxidation state and resulting covalency of the Co–O bond was observed for the series of solid solution cobaltites (La_1–*x*_Sr_*x*_CoO_3_).^10,11^ An increased Co oxidation state is consistent with the VBM values that are closer to the Fermi level for the bilayer films compared to the VBM of single LCO.

Thus, nanoscopic hybrid epitaxial bilayer films enable us to tune the surface covalency and the carrier concentration as a systematic transition between their two parent compounds.

To directly correlate covalency with the catalytic performance, we first have to clarify if band bending might influence the catalytic performance as well. To this end, we performed a systematic electrochemical characterization in combination with an MS analysis and compared its results with the obtained OER overpotentials.

### OER Activity Testing

**Figure 3:**
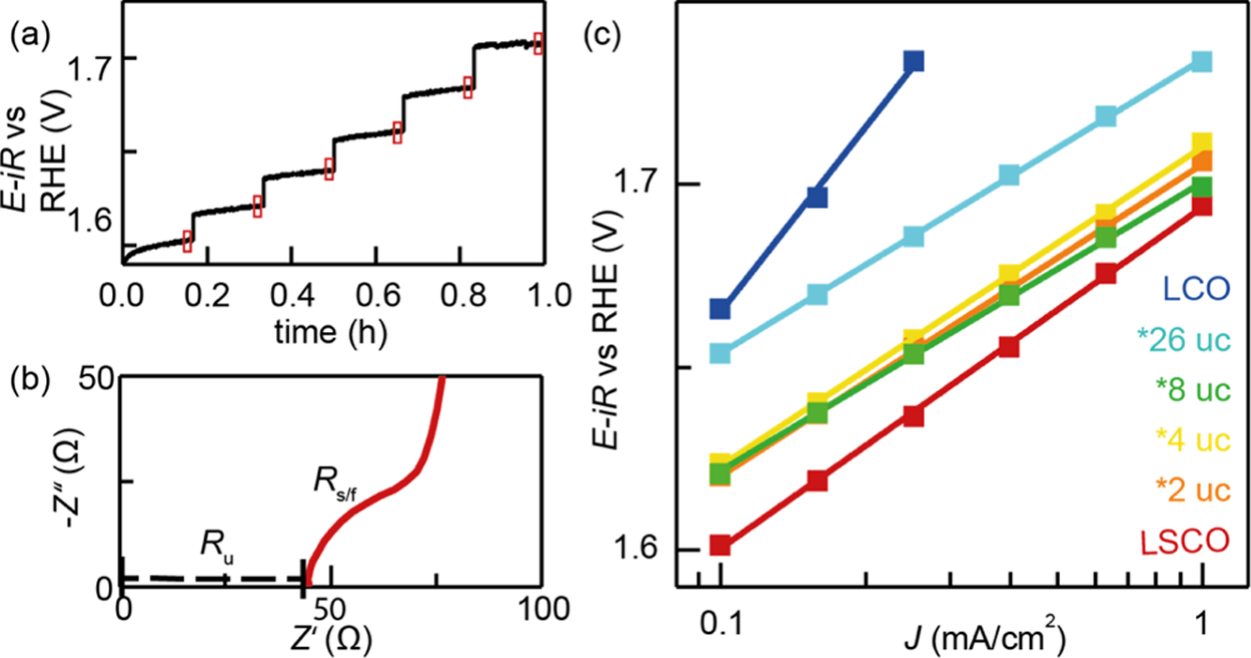
(a) Staircase CP of LSCO to determine the Tafel plot. The red boxes mark the last minute of each current step. In this range, the applied voltage was averaged to determine the mean overpotential for the six data points in the Tafel plot in Figure 3c. (b) Nyquist plot of an NSTO/LSCO film. The thin films were *iR*-corrected with the *R*_u_ and additionally with the NSTO/LSCO substrate/thin film interfacial resistance (*R*_s/f_). (c) Tafel plot of LSCO, LCO, and their bilayer films. The single LCO film results could not be corrected by the high NSTO/LCO resistance (*R*_s/f_).

The single and bilayer films of LSCO and LCO were tested in their OER catalytic activity by staircase chronopotentiometry (CP) where an exemplary plot is shown in Figure 3a. This approach minimizes the influence of additional currents (compared to typical cyclic voltammetry activity testing) unrelated to the OER such as capacitive charging or oxygen intercalation into the perovskite lattice.^3^ All samples were *iR*-corrected with the uncompensated resistance (*R*_u_) obtained from the *x*-axis offset in the Nyquist plot shown in Figure 3b and additionally by the resistance occurring from the NSTO/LSCO substrate/film interface (*R*_s/f_) represented by the semicircle marked in the impedance spectrum (the equivalent electric circuit is shown in Figure S8 and the impedance fit values are shown in Table S1). An exception from this is the single LCO film since the LCO/NSTO interfacial resistance exhibited a much higher value (*R*_s/f_ = 585–642 Ω compared to the typical values of LSCO/NSTO *R*_s/f_ = 21–71 Ω) and the *iR* correction including *R*_s/f_ led to an overcompensation of the measured potential (see Figure S9). The NSTO/LCO interfacial resistance seems to be highly voltage-dependent above 1.6 V vs RHE and was therefore not included in the *iR* correction. Thus, the LCO activity will only be evaluated at low current density values where the relative error due to incomplete *iR* correction remains small.

In Figure 3c, the Tafel plots obtained from the staircase CP measurements (as shown in Figure 3a) are shown. The single LSCO film shows the lowest required overpotential η = 380 mV at a current density of 0.1 mA/cm^2^, making it the most active catalyst in this sample series. The additional ultrathin LCO capping layers between 2 and 8 uc lead to an overpotential increase of ∼20 mV at 0.1 mA/cm^2^ compared to the single LSCO film. The thickest LCO bilayer film (26 uc LCO) and single LCO film exhibit the highest required overpotentials, with an increase of 50 and 60 mV compared to the single LSCO film (note that the overpotential for the single LCO film may be slightly overestimated as it was *iR*-corrected without *R*_s/f_ and should be similar to the 26 uc sample, see Figure S9). Therefore, an increase in the LCO thickness leads to an overpotential increase in the OER for the bilayer films with the trend of η(LSCO) < η(2, 4, and 8 uc LCO) < η(26 uc LCO and single LCO). Small deviations in the Tafel slope with typical values between 80 and 90 mV/dec (excluding the single LCO film slope) are observed. This also affects the overpotential trend for current densities above 0.1 mA/cm^2^ which may originate from the experimental error or differences in microkinetic mechanisms, voltage-dependent resistances, and degradation processes, which have increased impact at high current densities and will remain the subject of future research.

Overall, the bilayer sample of 26 uc LCO capping thickness appears to exhibit single LCO-like behavior, whereas the bilayer samples between 2 uc and 8 uc LCO capping thickness possess intermediate catalytic activity between LSC and LCO. The buried LSCO layer seems to activate the ultrathin LCO capping layers with negligible OER activity differences between 2 uc and 8 uc. The small overpotential differences between the samples with 2–8 uc LCO capping layer thickness are within the standard deviation range. A table with the observed OER overpotentials and relevant standard deviation is provided in Table S2.

The OER activity observed for the bilayer catalysts may stem either from the resulting Co–O covalency in the near surface (cf. Figure 2) or also from an increasing width of the surface space charge layer due to the more insulating character of LCO, as addressed below.

### Mott–Schottky Analysis

Next, we will discuss the influence of the band-bending conditions at the solid/liquid interface on the observed OER activity trend. The MS analysis is applied to investigate whether the conduction band and the valence band are bent downward or upward in the OER voltage regime (above 1.5 V vs RHE) and hence if a depletion layer or hole accumulation is present for the p-type catalyst surface. The latter would be desired for the anodic half-cell reaction.^5,14^ After the MS analysis, we can distinguish between the two OER descriptors of covalency and band bending.

Intuitively, LSCO with a higher hole concentration and almost metallic character should have a narrower space charge layer after Fermi level equilibration due to shorter screening lengths compared to LCO and the LCO capping layers. However, the extent of a space charge layer and the band bending direction (upward/downward) also depends on the relative position between the two Fermi levels of the electrolyte and electrode before equilibration that may differ between LSCO and LCO.^26^ Furthermore, the actual band bending and bending direction is voltage-dependent. Therefore, the mere investigation of the band alignment in UHV (see XPS results in Figure 2) or at OCV does not give accurate insights into the surface band alignment under OER conditions.^14^ To gain insights about the direction of band bending toward the OER voltage regime, we conducted an MS analysis to obtain the evolution of the space charge capacitance under applied electrochemical bias from OCV to 1.5 V vs RHE.

We recorded impedance spectra under DC bias in 50 mV increments and calculated the space charge capacitance  from the imaginary part of the impedance *Z*″ at the chosen frequency ϑ = 0.1 Hz (see Figure S10).^28^ In the resulting MS plot, the relation between the inverse, squared areal capacitance  is plotted as a function of the DC bias.

**Figure 4:**
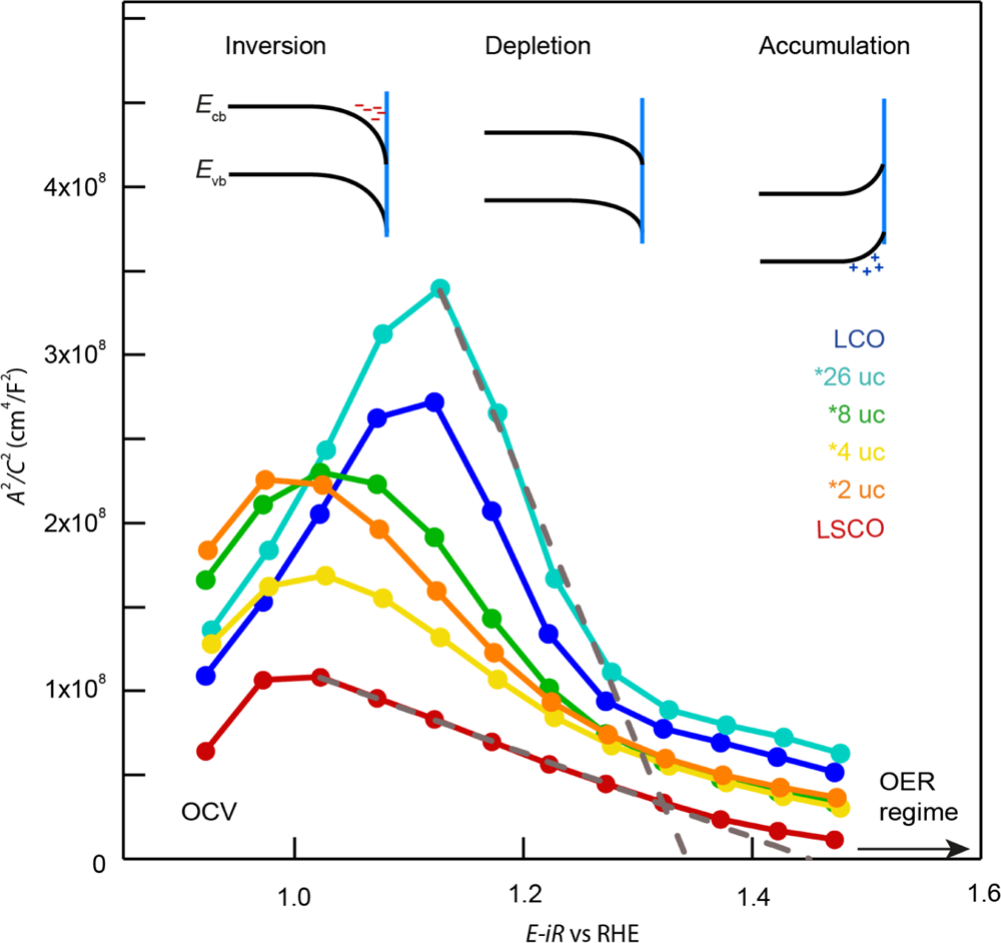
MS plots of single LSCO and LCO and bilayer films in alkaline media shown from OCV to the OER voltage regime. The dashed lines represent the linear extrapolation of the depletion region to obtain the flat band potential (between 1.35 and 1.45 V vs RHE for all samples). The corresponding scheme of conduction and valence band (CB and VB) bending as a function of the applied voltage is shown above.

The MS plots of the LSCO, LCO, and bilayer films are shown in Figure 4 with a representative band-bending model. Around OCV, we observe that all samples show n-type behavior (positive slope) indicating that the surface of the nominally p-type catalyst layers is in charge inversion due to downward band bending. With increasing potential, a transition from n-type to p-type behavior (negative slope) is evident between 1.0 and 1.2 V vs RHE, indicating a transition from charge inversion to a hole depletion region. The exact transition potential (maxima of the MS plots) depends on the LCO thickness. The single LCO film and bilayer film with 26 uc LCO capping thickness exhibit the lowest absolute space charge capacitance values at the maximum of the MS plot, consistent with the lowest surface doping concentrations.^29^ With further voltage increase, the extent of the depletion region is continuously decreased, whereas the flat band potential can be estimated from linear extrapolation of the MS plot, yielding 1.35 V vs RHE for the 26 uc LCO bilayer film (as well as for the single LCO film) and 1.45 V vs RHE for the LSCO film.^26^ Interestingly, the single and bilayer films show different slopes in the depletion region, consistent with a decreasing effective surface dopant concentration with increasing LCO thickness.^28,29^ Beyond the flat band potential, the MS plots flatten and possess comparable slopes indicating hole accumulation in the OER regime for all samples, which effectively provides sufficient electronic carriers to catalyze the OER.^5,13^

The overall trends in the MS analysis at OCV and low applied potentials are in line with the XPS analysis, revealing a decreased surface carrier concentration with increasing LCO thickness. Moreover, the inversion at OCV may even limit an electron transfer for oxidation processes.^5^ However, most strikingly, under OER conditions, all samples revealed hole accumulation and no OER limiting space charge barrier. Hence, it is unlikely that the electronic charge transfer at the electrolyte/catalyst interface suffers significantly from a lack of hole charge carriers under OER conditions for all samples. Therefore, we can rule out the initial relative position of the VBM observed for the hybrid bilayers and the downward band bending at OCV to be responsible for the reduced activity of LCO and LCO-capped catalysts. Instead, the hole accumulation above the flat band potentials may even enhance the OER. It is therefore apparent that the hybridization of LSCO and LCO and the resulting surface covalency indeed dominate the OER activity of the catalyst.

The resulting hybridization and surface Co–O covalency in the LSCO/LCO bilayer films up to 8 uc LCO capping thickness enhance the OER activity. In contrast, at an LCO capping thickness of 26 uc, the surface activation is diminished and LCO-like behavior is observed.

Prior studies found that the LOM reaction mechanism becomes thermodynamically favorable instead of the adsorbate evolution mechanism (AEM) in La_1–*x*_Sr_*x*_CoO_3_ with increasing Sr content.^10,11^ On LCO, only the AEM takes place where Co is the adsorption site for the OH^–^ ions. With increasing Sr content, the major adsorption site switches to lattice oxygen and the LOM takes place.^10,11^ As we observe intermediate OER activity for the bilayer films (2–8 uc), the resulting surface covalency in the LCO capping layer might also induce a change in the reaction mechanism to the LOM. The lower thermodynamic barriers in LOM compared to the AEM can be one reason for the decreased overpotential compared to a single LCO film. Therefore, covalency and actual OER mechanism go hand-in-hand suggesting that the bilayer geometry used here to tune the level of covalency in the catalysts may also allow tuning the actual OER mechanism by combining nanoscopic LSCO and LCO layers, which is an avenue for future research.

## Conclusions

For the design of perovskite oxide electrocatalysts for future applications, it is mandatory to understand, experimentally identify, and distinguish the material-dependent relevant OER descriptors. By employing a combined XPS and MS analysis, we experimentally distinguish here between the two possible OER descriptors, band bending at the solid/liquid interface and the covalency of the transition metal–oxygen bond in cobaltite thin films. The bilayer system of the two cobaltites LSCO and LCO enabled us to understand that the OER activity of the less-conductive and less-covalent LCO is limited by the larger Co 3d and O 2p separation and hence by the lower covalency and not by a limiting space charge layer at the catalyst surface under OER conditions.

The band bending in the OER voltage regime is characterized by hole accumulation at the surface for LSCO, LCO, and their bilayer films. This hole accumulation may even enhance the catalytic activity for the films, while a decreased surface doping concentration with increased LCO thicknesses in the bilayer systems does not lead to an undesired extended hole-depleted space charge region under operating conditions. Therefore, the covalency was found to dominate the OER activity in the La_1–*x*_Sr_*x*_CoO_3_ electrocatalysts, while the influence of space charge layers at the solid/liquid interface were found to be neglectable under OER conditions. For the bilayer films with ultrathin LCO capping layers from 2 to 8 uc on 52 uc LSCO, the OER activity showed intermediate behavior between LSCO and LCO.

In this way, we showed that combining nanoscopic perovskite layers yields interesting catalyst model systems to understand and tune the catalytic properties via surface electronic structure modifications, enabling us to systematically differentiate between possible OER descriptors such as covalency and band bending under operating conditions.

## Experimental Section

### Thin Film Fabrication

Epitaxial La_0.6_Sr_0.4_CoO_3_ and LaCoO_3_ films were deposited by PLD (TSST, B.V., Netherlands), using ceramic targets (Toshima Manufacturing Co., Ltd., Japan) and single crystalline 0.5 wt % niobium-doped SrTiO_3_ substrates with a KrF excimer laser (λ = 248 nm). The epitaxial growth was conducted with a target-to-substrate distance of 60 mm, a fluence of 2.2 J/cm^2^ (single LCO 2.4 J/cm^2^), a growth temperature of 650 °C (single LCO 600 °C), and an oxygen pressure of 0.053 mbar. The two-dimensional growth was tracked by RHEED. The pulse repetition rate was 5 Hz for the 52 uc-thick LSCO and LCO films. For the LCO upper layers of 2, 4, and 8 uc, the repetition rate was decreased to 1 Hz to ensure controllability of the deposition rate. For the subsequent LCO deposition on LSCO, the average LCO unit cell growth rate was taken from the first observed oscillations to set the required deposition time. Since the roughened surface of the buried 20 nm-thick LSCO layer did not always allow appropriate oscillation tracking for the subsequent LCO growth, the deposition time was set to the average LCO growth rate seen from previous LCO samples grown under the same PLD conditions.

### Physical Characterization

The surface morphology was scanned with the atomic force microscope Cypher SPM (Research Asylum, Germany) in the tapping mode. The tips were provided by NanoWorld AG (Switzerland) made out of silicon. Crystallographic properties were obtained by XRD with a D8 ADVANCE diffractometer (Bruker AXS GmbH, Karlsruhe, Germany) which is equipped with a monochromized Cu K_α1_ radiation source.

XPS was performed with a Phi 5000 VersaProbe II system (ULVAC Phi, Physical Electronics Inc., USA) with Al K_α_ X-ray illumination without charge neutralization. The pass energy was 23.5 eV for the Co 3d and La 3d spectra and 188 eV for the Sr 3d spectra to determine the surface stoichiometry of the films in reference to the LSCO and LCO target composition. The Shirley background was subtracted from the data in the Casa XPS software. The pass energy for the valence band spectra was 47 eV. The photoemission angles were θ = 15° and θ = 55°, which corresponds to a mean escape depth of *d* = 2.6 nm and 1.6 nm. *d* was calculated via *d* = λ × cos θ,^30^ where λ is the inelastic mean free path of the photoelectrons, λ = 2.77 nm calculated for LSCO and LCO with the software QUASES-IMFP-TPP2M.^31^ The energy scale of the instrument was calibrated and confirmed on an Au foil before and after the set of measurements. The linear combination model for the bilayer films out of the raw LSCO and LCO spectra was calculated with a principal component analysis via the Casa XPS software. The original spectra were normalized at 11 eV before the linear combination was conducted.

### Electrochemical Characterization

The samples were placed in a three-electrode electrolyzer rotating disk electrode (RDE) setup which was connected to a BioLogic SP-150 potentiostat (Bio-Logic Science Instruments, France). A Hg/HgO electrode (CH152 by CH Instruments, USA), which was calibrated to the reversible hydrogen electrode (HydroFlex, USA) in 0.1 M KOH with typical values of ∼880 mV, served as the reference electrode and a platinum wire as the counter electrode. The drift of the reference electrode lies within 10 mV. To ensure contact of the sample in the RDE setup, the whole back and side walls as well as the edges on the front side of the sample were coated with a 50 nm Pt layer by sputtering and then placed at the tip of the rotary shaft using a custom-made PEEK adapter with an O-ring (FFKM, ERIKS, Germany) with 0.75 cm diameter (a sample sketch is shown in Figure S11). The electrodes were immersed in O_2_-saturated (30 min O_2_ supply aforehand) 0.1 M KOH solution prepared by dissolving KOH pellets (Sigma-Aldrich, 99.99%) in deionized water (Milli-Q, >18.2 MΩ cm). All electrochemical experiments were conducted with a rotation rate of 1600 rpm. The entire RDE setup was placed under a nitrogen/oxygen atmosphere in a glovebox. Staircase CP measurements were kept at each step constant for 10 min with chosen current density steps at 0.1, 0.160, 0.250, 0.397, 0.631, and 1.0 mA/cm^2^ to determine the OER activity and the Tafel plot under steady-state conditions.^3^ The potential values of the last minute in each 10 min step were averaged to calculate one potential value for each current density step.

Impedance spectroscopy was taken at OCV with a sinusoidal voltage of 20 mV at frequencies of 0.1 Hz to 1 kHz to accurately *iR*-correct the OER activity data. First, the Ohmic uncompensated resistance *R*_u_ was determined by the high-frequency intercept of the real impedance using a linear fit. Second, the substrate/thin film interfacial Schottky resistance *R*_s/f_ caused by the n-type NSTO substrate and the p-type LSC/LCO catalyst layer was determined by an impedance fit in the EC-Lab software (Bio-Logic Science Instruments, France). For the MS analysis, the impedance was measured at each DC voltage step from 0.05 to 0.6 V vs Hg/HgO with an increase of 50 mV and in the frequency range of 0.1–20.000 Hz. The best match for the accurate capacitance determination at a single frequency was found for 0.1 Hz confirmed by an impedance *z*-fit in the EC lab software. The applied equivalent electric circuit can be seen in Figure S8.

## Supporting Information Available

AFM scans of LSCO and LCO, deposition tracking of bilayer samples with RHEED, RHEED pattern before and after deposition, XAS Co K-edge data measured in the fluorescence detection mode, component fitting of XPS valence band spectra for LSCO and LCO, linear combination model of LSCO and LCO XPS spectra to identify additional hybrid electronic states, linear combination model of XPS spectra with different photoemission angles and bilayer thicknesses, equivalent electric circuit, table of impedance fit for *iR* correction, Nyquist plot substrate/thin film interfacial resistance for LCO and *iR* correction, table of OER potentials at 0.1 mA/cm^2^ with the standard deviation, example of staircase electrochemical impedance spectroscopy data for the MS analysis, and sample sketch of the epitaxial film sputtered Pt contacts and how it is covered with the O-ring (PDF)
