# Supplementary material for: Separating the Effects of Band Bending and Covalency in Hybrid Perovskite Oxide Electrocatalyst Bilayers for Water Electrolysis
Source: ACS Appl Mater Interfaces. 2022 Mar 16;14(12):14129–36. doi: 10.1021/acsami.1c20337 (PMC8972245; doi:10.1021/acsami.1c20337)
Supplement: Supplementary file 1 — am1c20337_si_001.pdf [file am1c20337_si_001.pdf]

# Supporting information

## Separating the effects of band bending and covalency in hybrid perovskite oxide electrocatalyst bilayers for water electrolysis

**Lisa Heymann,**[†*] **Moritz L. Weber,**[†] **Marcus Wohlgemuth,**[†] **Marcel Risch, Regina Dittmann,**[†] **Christoph Baeumer,**[†,§*] **Felix Gunkel**[†*]

[†]Peter Gruenberg Institute 7, Forschungszentrum Juelich GmbH, 52425 Juelich, Germany and JARA-FIT, RWTH Aachen University, 52056 Aachen, Germany

[#]Nachwuchsgruppe Gestaltung des Sauerstoffentwicklungsmechanismus, Helmholtz-Zentrum Berlin für Materialien und Energie GmbH, 14109 Berlin, Germany

[§]MESA+ Institute for Nanotechnology, Faculty of Science and Technology, University of Twente, Enschede, Netherlands

[*]E-mail address of corresponding authors: Lisa Heymann (l.heymann@fz-juelich.de), Christoph Baeumer (c.baeumer@utwente.nl), Felix Gunkel (f.gunkel@fz-juelich.de)

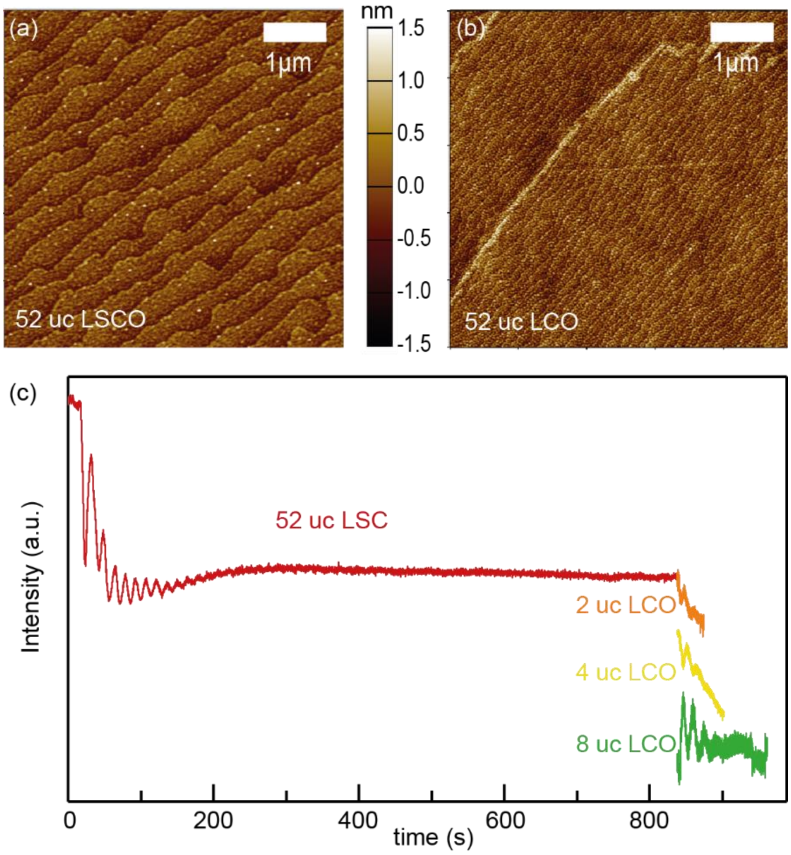


**Figure S1**: AFM scan of an a) 52 uc LSCO and b) 52 uc LCO film. c) Bilayer deposition for LCO capping layers in thicknesses of 2, 4, 8 uc.

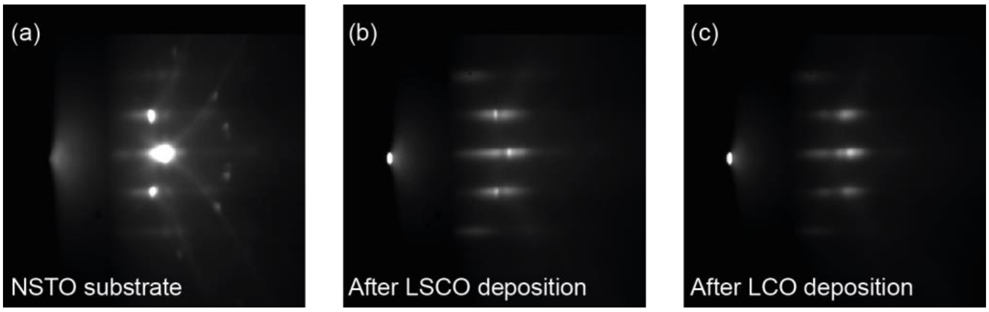


**Figure S2**: RHEED pattern of **a)** the bare NSTO substrate with the specular spot and corresponding diffraction spots appearing on the Laue circle of a two-dimensional surface **b)** a 52 uc LSCO thin film surface **c)** a 52 uc LSCO/8 uc LCO film with further broadened spots, indicative of a two-dimensional, slightly roughened surface.

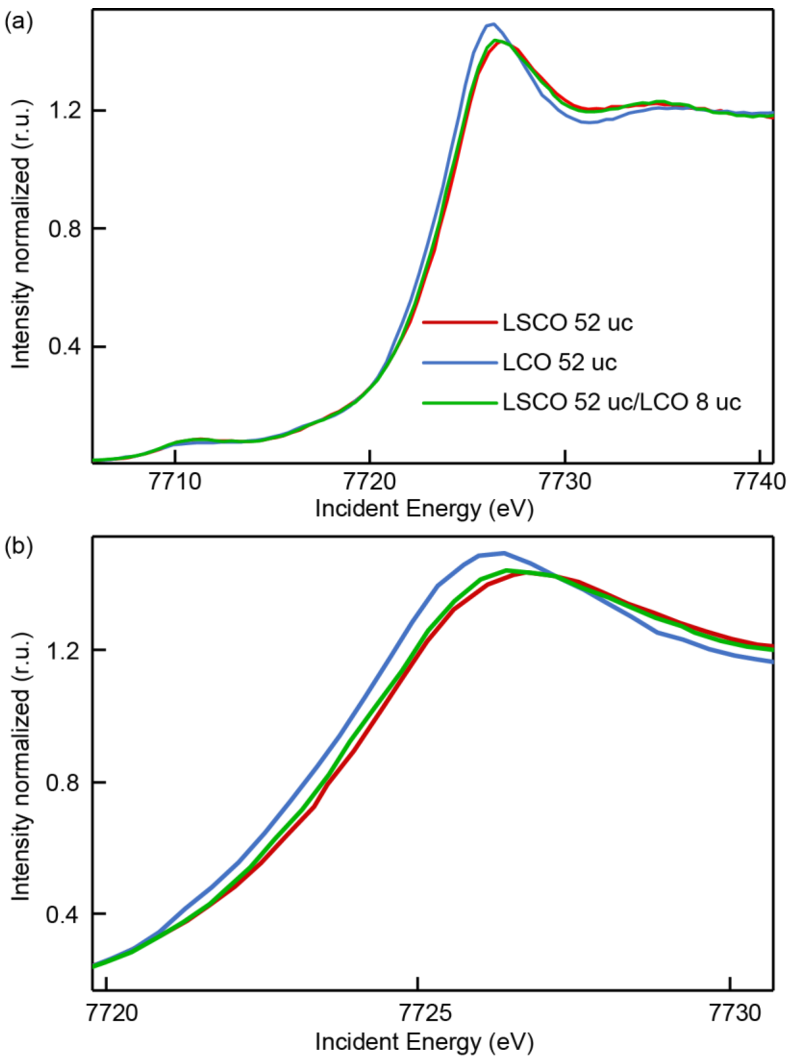


**Figure S3:** (a) XAS data of the Co K-edge with pre-edge are shown for a 52 uc thick LSCO and LCO film as well as for a 52 uc LSCO/8 uc LCO bilayer film measured in fluorescence detection mode. (b) Zoom to the Co K-edge of the same data as in (a) to observe the energy differences of the absorption edges.

**Experimental setup XAS:** X-ray absorption spectroscopy at Co K-edge was performed at the CryoEXAFS endstation[1] on the KMC-3 beamline at the BESSY II electron storage ring operated in top-up mode at 300 mA. The spectra were recorded in fluorescence mode using a 13-element silicon drift detector (SDD) from RaySpec. A Si (111) double-crystal monochromator and a horizontally polarized beam were used. The energy was calibrated using a Co metal foil (fitted reference energy of 7709 eV in the first derivative spectrum) with an accuracy ±0.1 eV. At least four scans of each sample were collected to k = 14 $Å^{-1}$. All spectra were processed by correcting for the detector dead time and division of the incident beam intensity measured by an ion chamber. The spectra were further normalized by subtracting a constant obtained by fitting the data before the K-edge and division by a $2^{nd}$ order polynomial function obtained by fitting the data after the K-edge. The attenuation length of the fluorescence at the averaged Co $K_{\alpha}$ emission (6922.8 eV) in $La_{0.6}Sr_{0.4}CoO_3$ (density 6.7 g/cm$^3$) at 45°

incidence was calculated as 4.3 µm using the Henke tables.[2] The attenuation length in $LaCoO_3$ (density 6.9 g/cm$^3$) was calculated as 3.2 µm using the same parameters. We estimate the escape depth as trice the attenuation length, which is at least 12.9 µm, i.e., much larger than the $La_{1-x}Sr_xCoO_3$ layer(s) of 20-23 nm.

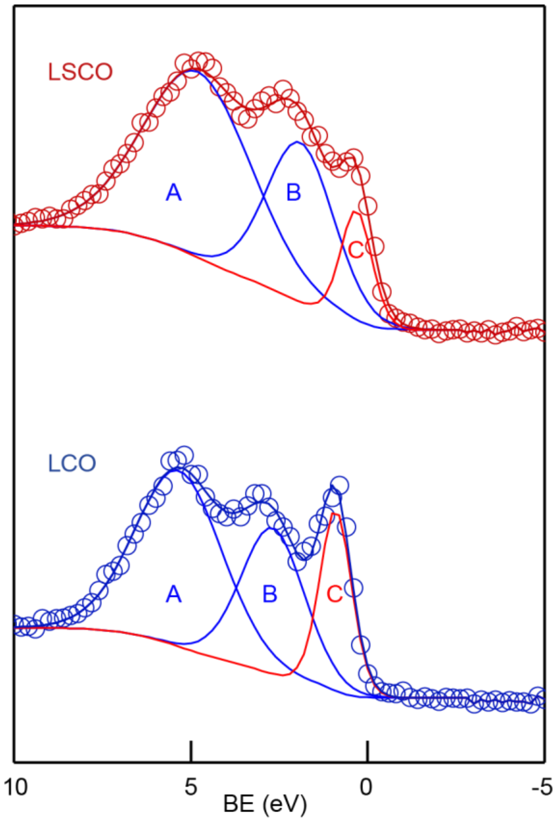


**Figure S4:** Fitting of the LSCO and LCO valence band spectra, conducted for the single thin film samples: The original valence band XPS spectra of the LSCO and LCO film are shown as open circles and the envelope of the component fit are drawn as solid lines. The three components are A and B for the O 2*p* states and C for the Co 3*d* states. A Gauss-Lorentz peak shape with a 40:60 ratio was chosen. The distance of the two maxima of B and C is only 1.6 eV for LSCO and 1.8 eV for LCO. The FWHM of peak B is 2.26 eV for LSCO and only 2.11 eV for LCO. The peak position and width determine the total O 2*p*-Co 3*d* overlap, which is smaller for LCO than for LSCO.

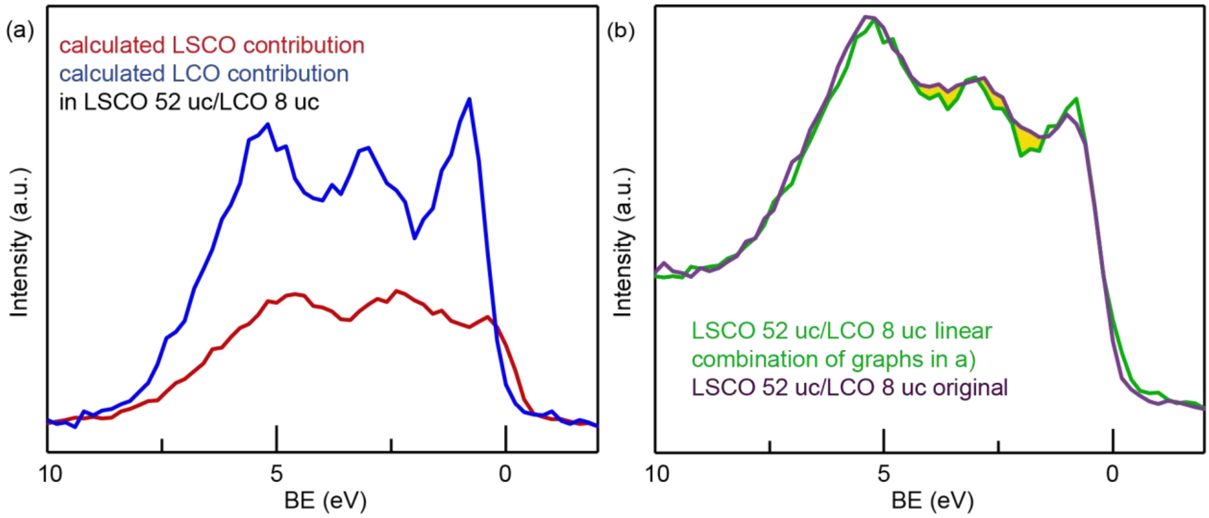

**Figure S5**: **a)** linear combination results of the valence band electronic structure, calculated from the Casa XPS software for the 52 uc LSCO/8 uc LCO sample: the shape of the spectra stems from the original LSCO and LCO XPS valence band spectra; the intensity was calculated from the casa XPS software in evaluation of the total contribution of each spectrum to the 52 uc LSCO/8 uc LCO spectrum. The Shirley background was subtracted. **b)** 52 uc LSCO/8 uc LCO XPS original spectrum of the valence band electronic structure is shown together with its calculated linear combination from LSCO and LCO in a).

As can be seen from the filled yellow area, the linear combination underestimates the density of states in the overlapping region of the O 2*p* and Co 3*d* bands. Therefore, the bilayer sample exhibits a higher covalency than expected from the linear combination from the LSC and LCO single spectra. The presence of the buried LSCO film increases the surface LCO covalency. A detailed linear combination analysis can be seen for two different photoemission angles in S6.

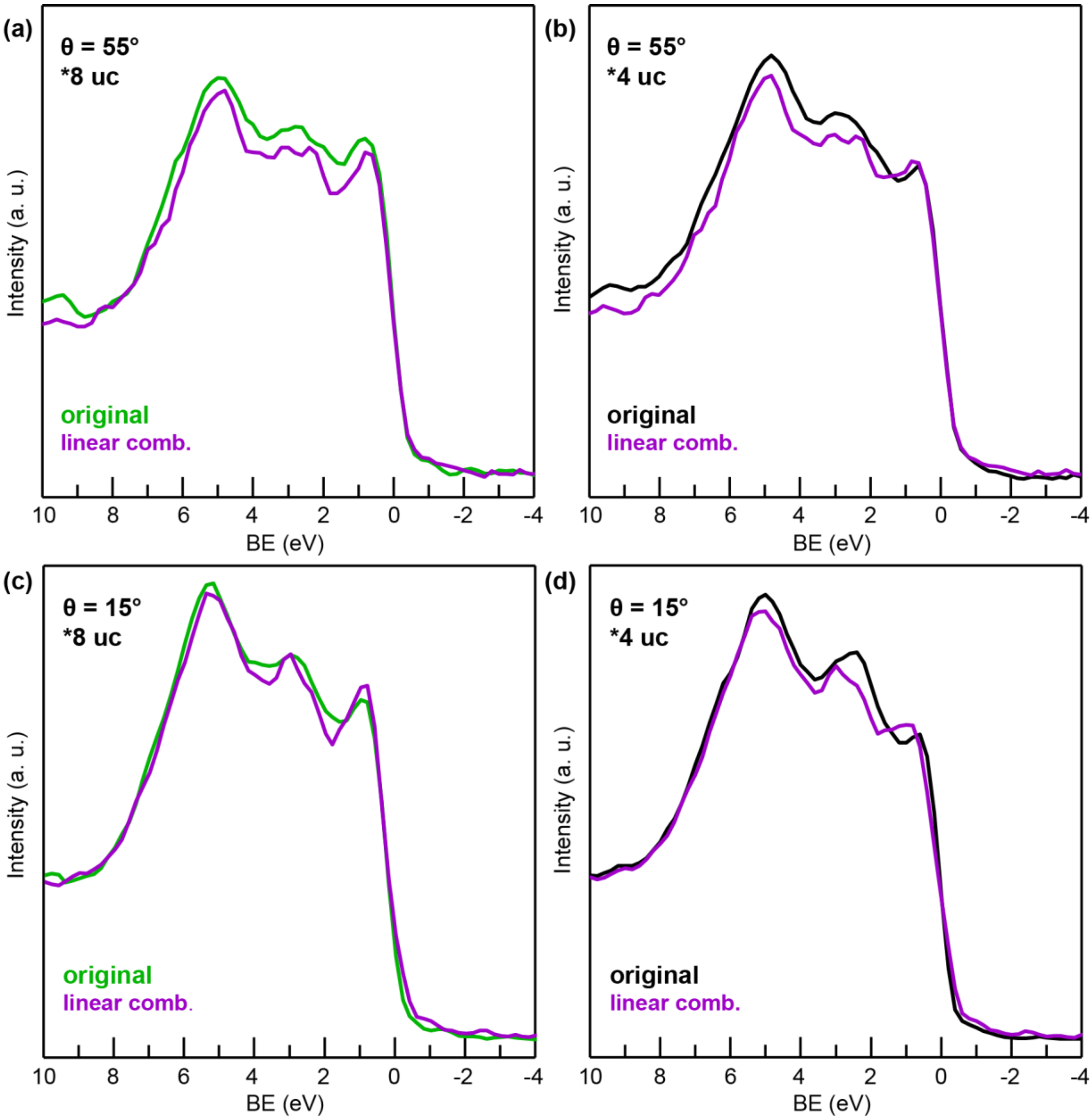


**Figure S6:** The linear combination results (purple lines) and the corresponding original valence band spectra are shown in (a) and (b) with a photoemission angle of θ = 55° (*d* = 1.6 nm) for the *8 uc and *4 uc bilayer film respectively (the bilayer films are marked with a *). In (c) and (d) the spectra with θ = 15° (*d* = 2.6 nm) are shown for the *8 uc and *4 uc bilayer films. The XPS spectra with θ = 55° exhibit a poorer signal to noise ratio and therefore a Savitzky Golay algorithm over 5 points was used to smooth the XPS spectra. For comparison we also smoothed the data at θ = 15° for this figure.

Comparing two mean escape depths *d* aims to observe if there are different LCO covalency contributions depending on the different LSCO signal contributions. Overall, a reasonable match between the linear combination and the measured (smoothed) spectra is apparent, with a better match for θ = 15°. However, the linear combination result systematically underestimates for all spectra the density of states that exist in the original spectra at around 1-4 eV (peak B). A small

underestimation is also seen for peak A between 4-6 eV. The O 2*p* peak B (1-4 eV) appears to be wider than expected from a mere linear-combination scenario, seen in the spectra of both photoemission angles. The larger width of the O 2*p* peak (B) results in a higher overlap with the Co 3*d* states in the raw data compared to the linear combination model. Therefore increased surface covalency is apparent in the thin LCO capping layers. The XPS spectra hence exhibit a superposition of the LSCO and LCO layer and additionally hybrid states. Importantly, for the more surface sensitive spectrum with a mean escape depth of 1.6 nm (θ = 55°) the pure LSCO layer contribution is lower and therefore the spectra contain higher LCO character, but even more prominently exhibit increased covalency compared to the linear combination.

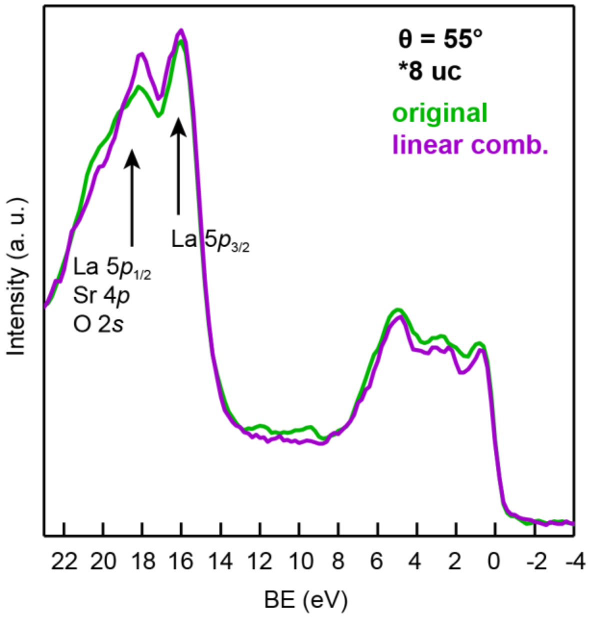


**Figure S7:** The XPS spectrum and according linear combination result of Figure S6a of the *8 uc bilayer sample is shown with an extended binding energy range from 23 eV to -4 eV. In the range from 14-22 eV the La 5$p_{3/2}$ core level peak is observed at 16 eV and above 16 eV the La 5$p_{1/2}$, Sr 4*p* and O 2*s* are observed.

Especially in Figure S6a the density of states below 10 eV appears to be in general underestimated from the linear combination compared to the original spectrum. This is not a result of inaccurate normalization of the spectra as confirmed in the extended spectrum range shown in Figure S7: The strontium (Sr 4*p* at around 18 eV) contribution is underestimated from the linear combination and the La contribution (La 5$p_{3/2}$ (16 eV)) has similar peak intensity for the raw data and the linear combination. According to the Sr underestimation but reasonable match of La contribution, we conclude that the generally underestimated density of states below 10 eV are the result of the hybrid states in the LCO capping layers and not an inaccurate normalization of the data. Additionally, the underestimated Sr

contribution emphasizes that the increased Co 3*d*-O 2*p* overlap in the original spectra does not only stem from a superimposed LSCO signal but from hybrid states that increase the Co 3*d*-O 2*p* overlap in the LCO capping layers.

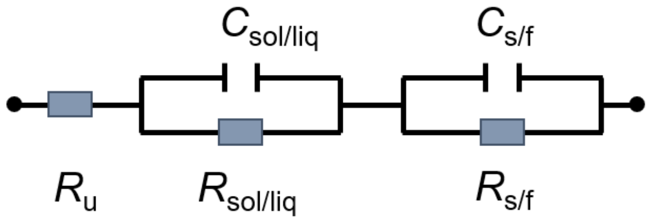


**Figure S8**: equivalent electric circuit applied for the *iR* correction and Mott-Schottky analysis. $R_u$ is the uncompensated resistance, $C_{sol/liq}$ and $R_{sol/liq}$ element stem from the solid/liquid interface and $C_{s/f}$ and $R_{s/f}$ stem from the substrate/thin film interface. For an accurate impedance fit the interfacial capacitance of the solid/liquid interface was replaced by a constant phase element *Q* in the equivalent electric circuit.

**Table S1:** The table lists the calculated $R_u$ and $R_{s/f}$ from the impedance fit. The values were used to *iR* correct the stair case CP data. The impedance spectra were recorded at OCV. For the purpose of optimizing the fit, the $C_{sol/liq}$ was replaced by the constant phase element $Q_{sol/liq}$ revealing an ideality factor *a* close to unity.

| **Element** | **unit** | **LSCO** | ***2 uc** | ***4 uc** | ***8 uc** | ***26 uc** | **LCO** |
|---|---|---|---|---|---|---|---|
| $R_u$ | Ω | 45 | 43 | 46 | 47 | 45 | 44 |
| $Q_{sol/liq}$ | $F.s^{a-1}$ | 2.86E-05 | 2.71E-05 | 2.95E-05 | 2.82E-05 | 3.10E-05 | 3.01E-05 |
| ***a* for Q** | - | 0.97 | 0.98 | 0.94 | 0.99 | 0.95 | 0.95 |
| $R_{sol/liq}$ | Ω | 1.34E+05 | 2.20E+05 | 1.59E+06 | 2.01E+05 | 8.19E+05 | 1.04E+06 |
| $R_{s/f}$ | Ω | 30 | 21 | 51 | 25 | 72 | 585 |
| $C_{s/f}$ | F | 4.92E-06 | 4.43E-06 | 5.80E-06 | 5.07E-06 | 4.51E-06 | 6.59E-06 |

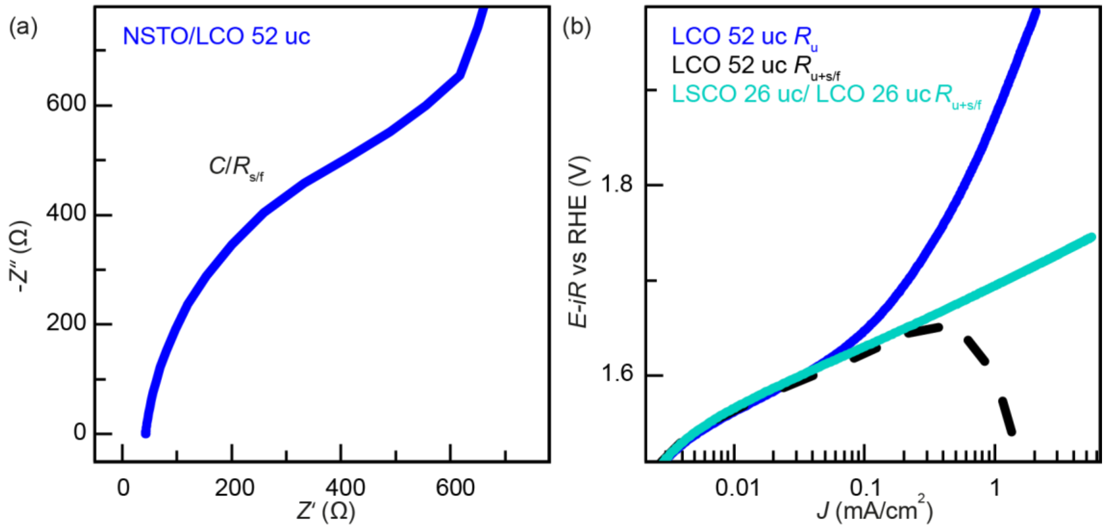


**Figure S9**: **a)** Nyquist plot in the high frequency range of the NSTO/LCO 52 uc film where the observed semicircle corresponds to the *C*/*R* element of the substrate/thin film interface $C_{s/f}$ and $R_{s/f}$

**b)** the averaged cyclic voltammetry (CV) scans are shown for the NSTO/LCO 52 uc film

1) in blue with an *iR* correction including only $R_u$,

2) in black dashed lines with $R_u$ and additional $R_{s/f}$ correction which leads to the overcompensation of the plot.

3) the CV scan in light blue of the sample NSTO/LSCO 26 uc/LCO 26 uc is shown where the $R_u$ and $R_{s/f}$ correction was conducted and shows a neither over- nor undercompensated OER behavior.

As the single NSTO/LCO film and the bilayer film NSTO/26 uc LSCO/26 uc LCO show remarkably similar overpotentials up to 1.6 V vs RHE they exhibit the same OER catalytic activity and the deviation at higher voltages is stemming from the different substrate/thin film resistances ($R_{s/f}$) and not from another intrinsic catalytic behavior.

**Table S2:** The OER potential at 0.1 mA/cm$^2$ is shown for all samples with the corresponding standard deviation. The bilayer films of 2, 4 and 8 uc have small potential differences that lie in the experimental error (the bilayer films are marked with a *).

| Sample | Potential (V vs RHE) at 0.1 mA/cm$^2$ |
|---|---|
| LSCO | 1.601 ± 0.01 V |
| *2 uc | 1.620 ± 0.01 V |
| *4 uc | 1.623 ± 0.01 V |
| *8 uc | 1.622 ± 0.01 V |
| *26 uc | 1.654 ± 0.01 V |
| LCO (only $E\text{–}iR_u$) | 1.666 ± 0.01 V |

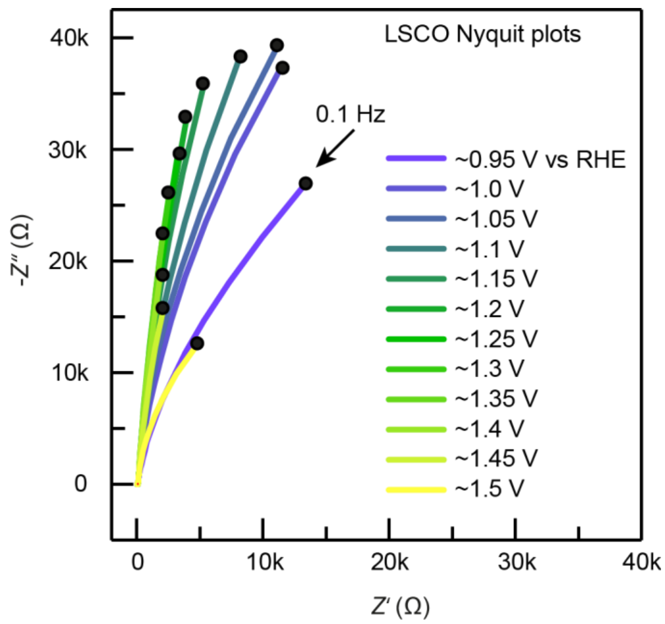


**Figure S10**: Nyquist plots of an NSTO/LSCO 52 uc film at stepwise increased bias from about 0.95 V to 1.5 V vs RHE. The beginning of the large semicircle represents the $C_{s/f}/R_{s/f}$ element of the solid/liquid interface that is voltage dependent towards the OER regime. The most accurate fitting results to determine the Mott-Schottky plot (Capacitance mainly measured at the solid/liquid interface) were achieved with the imaginary part of the impedance at 0.1 Hz which was confirmed by a z fit simulation of the entire frequency space with the applied equivalent electric circuit seen in Figure S8 calculated via the software EC-Lab® (*Bio-Logic Science Instruments*, France).

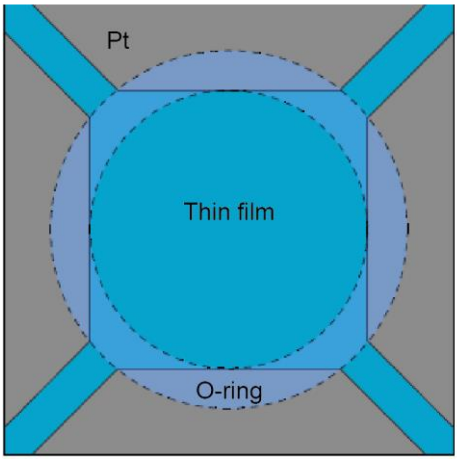


**Figure S11**: an exemplary sample sputtered with Pt lashes along the edges on the thin film front side, the two circles in dashed lines represent the O-ring that isolates the contacts in the electrolyzer working electrode from the KOH solution

SI References

(1) Helmholtz-Zentrum Berlin Für Materialien Und Energie. (**2020**). CryoEXAFS: X-Ray Absorption Spectroscopy Station with Cryogenic or in-Beam Operando Electrochemistry Sample Conditions at BESSY II. *Journal of Large-Scale Research Facilities*, 6, A139. Http://Dx.Doi.Org/10.17815/Jlsrf-6-176.

(2) Henke, B. L.; Gullikson, E. M.; Davis, J. C. X-Ray Interactions: Photoabsorption, Scattering, Transmission, and Reflection at E = 50-30,000 EV, Z = 1-92. *At. Data Nucl. Data Tables* **1993**, *54* (2), 181–342. https://doi.org/10.1006/adnd.1993.1013.
